# Genome-wide association analysis on normal hearing function identifies *PCDH20* and *SLC28A3* as candidates for hearing function and loss

**DOI:** 10.1093/hmg/ddv279

**Published:** 2015-07-17

**Authors:** Dragana Vuckovic, Sally Dawson, Deborah I. Scheffer, Taina Rantanen, Anna Morgan, Mariateresa Di Stazio, Diego Vozzi, Teresa Nutile, Maria P. Concas, Ginevra Biino, Lisa Nolan, Aileen Bahl, Anu Loukola, Anne Viljanen, Adrian Davis, Marina Ciullo, David P. Corey, Mario Pirastu, Paolo Gasparini, Giorgia Girotto

**Affiliations:** 1 Department of Medical, Surgical and Health Sciences, University of Trieste, Trieste 34100, Italy,; 2 UCL Ear Institute, University College London, London WC1X 8EE, UK,; 3 Howard Hughes Medical Institute and Department of Neurobiology, Harvard Medical School, Boston, MA 02115, USA,; 4 Gerontology Research Center and Department of Health Sciences, University of Jyväskylä, Jyväskylä FI-40014, Finland,; 5 Institute for Maternal and Child Health IRCCS ‘Burlo Garofolo’, Trieste 34100, Italy,; 6 Institute of Genetics and Biophysics ‘A. Buzzati-Traverso’, CNR, Naples 80131, Italy,; 7 Institute of Population Genetics, National Research Council of Italy, Sassari 07100, Italy,; 8 Institute of Molecular Genetics, National Research Council of Italy, Pavia 27100, Italy,; 9 Department of Public Health, Hjelt Institute, University of Helsinki, Helsinki FI-00014, Finland and; 10 Experimental Genetics Division, Sidra, Doha, Qatar

## Abstract

Hearing loss and individual differences in normal hearing both have a substantial genetic basis. Although many new genes contributing to deafness have been identified, very little is known about genes/variants modulating the normal range of hearing ability. To fill this gap, we performed a two-stage meta-analysis on hearing thresholds (tested at 0.25, 0.5, 1, 2, 4, 8 kHz) and on pure-tone averages (low-, medium- and high-frequency thresholds grouped) in several isolated populations from Italy and Central Asia (total *N* = 2636). Here, we detected two genome-wide significant loci close to *PCDH20* and *SLC28A3* (top hits: rs78043697, *P* = 4.71E−10 and rs7032430, *P* = 2.39E−09, respectively). For both loci, we sought replication in two independent cohorts: B58C from the UK (*N* = 5892) and FITSA from Finland (*N* = 270). Both loci were successfully replicated at a nominal level of significance (*P* < 0.05). In order to confirm our quantitative findings, we carried out RT-PCR and reported RNA-Seq data, which showed that both genes are expressed in mouse inner ear, especially in hair cells, further suggesting them as good candidates for modulatory genes in the auditory system. Sequencing data revealed no functional variants in the coding region of *PCDH20* or *SLC28A3*, suggesting that variation in regulatory sequences may affect expression. Overall, these results contribute to a better understanding of the complex mechanisms underlying human hearing function.

## Introduction

The identification of new genes involved in modulating the auditory system will provide important insight into the complex mechanisms and the extensive heterogeneity of the hearing process. Although much effort has been made to search for genes involved in hearing function and loss, the molecular basis of normal hearing function is still largely unknown. For many complex traits, there is evidence that rare Mendelian mutations, copy number variation and rare variants contribute to a proportion of cases. More than 140 loci associated with non-syndromic hearing loss have now been mapped, and ∼80 genes have been identified in humans (http://hereditaryhearingloss.org/). So far, very few genes have been described as possibly involved in complex phenotypes (e.g. normal hearing function and age-related hearing loss). Quite recently, genome-wide association studies (GWAS) have produced a list of SNPs/genes showing association with these traits. In particular, a GWAS identified significant associations between age-related hearing impairment and a glutamate receptor *GRM7* in European populations (1), followed by a replication in a European American population (2). In our previous GWAS meta-analysis, using European isolated populations, we detected several suggestive associations (3). A later follow-up of these results highlighted 12 genes, which were replicated and validated at the expression level (4), including another glutamate receptor named *GRM8*. Furthermore, a recent GWAS meta-analysis identified *SIK3* (5), a member of the salt-inducible kinase family known to be involved in the inner ear. In a candidate gene study, the estrogen-related receptor gamma was suggested as a potential player in the maintenance of hearing in both humans (mainly females) and mice (6).

Considering recent findings and the extremely intricate mechanisms underlying the auditory process, we reasoned that GWAS might help in the detection of new genes involved in the variability of the hearing process; such genes may play a role in defining penetrance, progression, expressivity, pleiotropy and age of onset of hearing loss. We therefore performed a GWAS meta-analysis based on 1000 Genomes imputation data in several Italian isolated populations and identified 2 statistically significant candidates for modulating normal hearing function. These hits were then replicated in different communities located along the Silk Road and in two independent cohorts from UK and Finland.

## Results

### Statistical association

**Figure 1.**
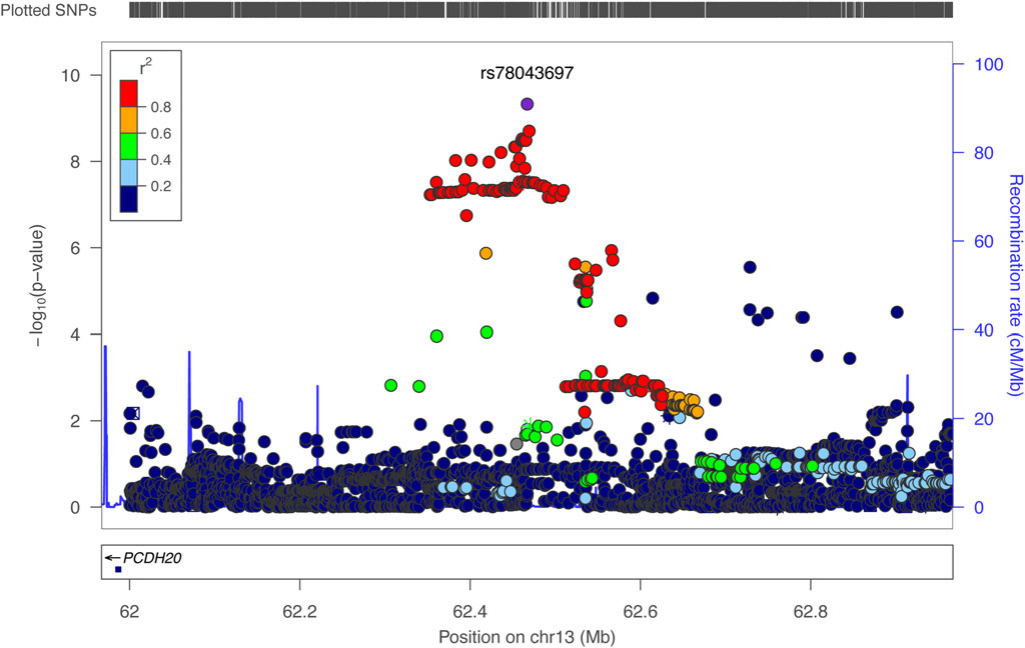
**Locus-zoom plot for chromosome 13 locus.** The plot shows the locus on chromosome 13 associated with normal hearing functions measured at 2 kHz, highlighting the top SNP rs78043697 and several other significant SNPs in the same region being in high linkage disequilibrium. The results plotted were obtained with a two-stage meta-analysis, focusing on Italian isolates first and then including the communities from the Silk Road. The *y*-axis shows −log_10_ of the *P*-values obtained from the complete meta-analysis of the 2 kHz hearing threshold. The closest gene *PCDH20* is shown along the *x*-axis.

**Figure 2.**
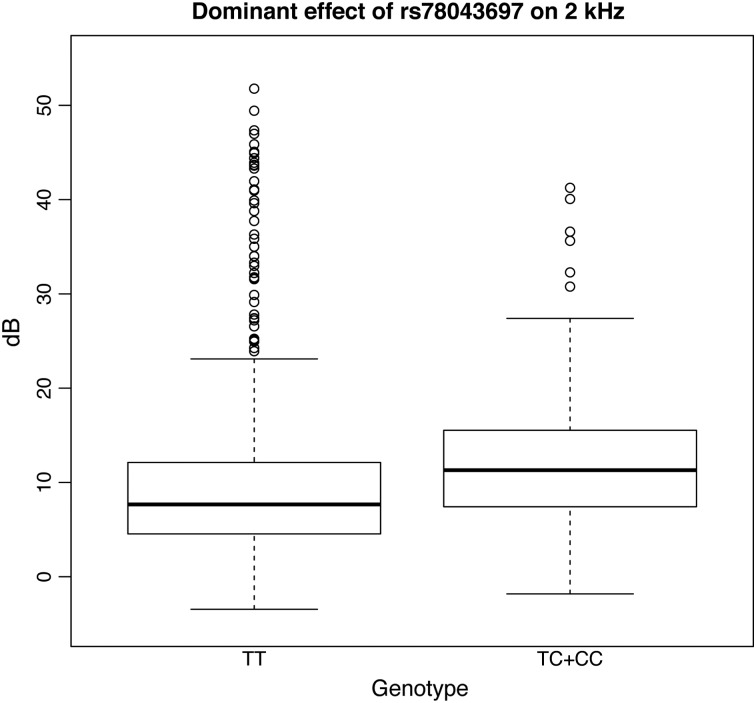
**Boxplot for rs78043697.** The figure displays boxplots for the 2 kHz frequency trait divided by genotype groups for rs78043697 and adjusted by sex, age and relatedness in our largest cohort (INGI-FVG). As the effect for this SNP was observed under a dominant model, the heterozygote genotype TC and the homozygote CC are shown together.

**Figure 3.**
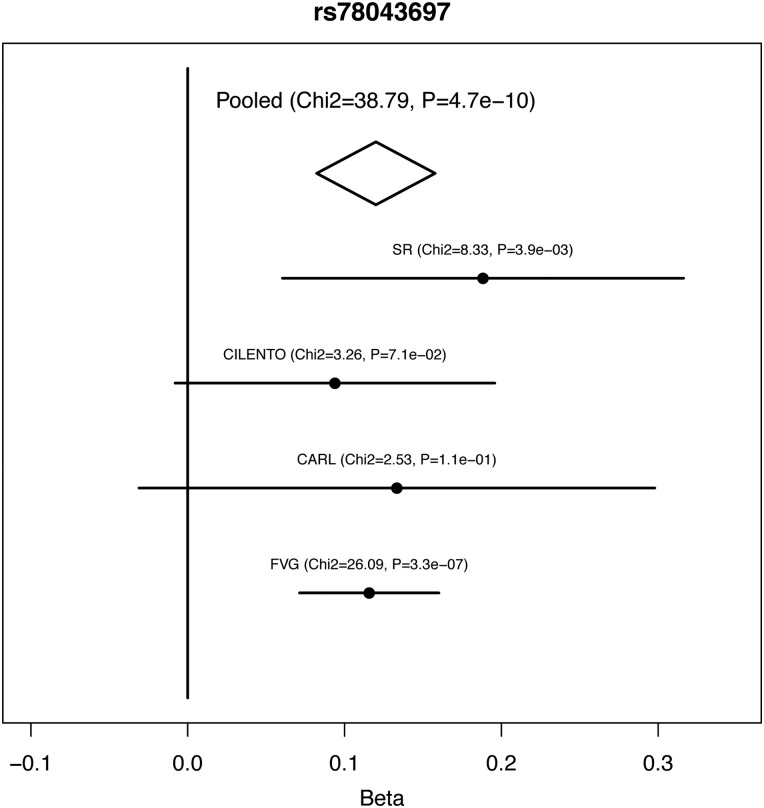
**Forest plot for rs78043697.** The figure shows the forest plot for the top SNP associated with 2 kHz threshold on chromosome 13 after performing the two-stage meta-analysis. The *x*-axis represents effect sizes (beta coefficients) for trait residuals after sex, age and kinship adjustments. The strongest association can be observed in the INGI-FVG cohort.

The first-stage meta-analysis was performed in 2155 subjects from 4 Italian cohorts, testing 9 hearing measurements (6 single frequencies and 3 PTAs). There were no signs of population stratification (all lambda coefficients ≤ 1.01), and a total of 223 SNPs reached a *P*-value of < 1e−06 (Supplementary Material, Table S1). These 223 SNPs were further analyzed by combining data from a completely independent cohort of isolated communities (Silk Road cohort, *N* = 481), resulting in a total sample size of 2636. In the combined analysis, 35 out of the 223 SNPs were replicated on the same traits and genetic models, reaching a genome-wide significant *P*-value of <5e−08 (Supplementary Material, Table S2). Among the 35 significant SNPs, 32 were located within 1 large locus on 13q21.31 (chr13:62382925–chr13:62520492 according to build 37) and located close to *PCDH20* (Fig. 1). The top SNP in this region is rs78043697-C, which reached a *P*-value of 4.71E−10 on the 2 kHz trait under the dominant model. Subjects homozygous for the non-effect allele T (*N* = 1964) have a better hearing compared with the other groups (*N* = 191), e.g. the difference between median values in INGI-FVG was 3.7 dB (Fig. 2). The remaining SNPs are in very high linkage disequilibrium with the top SNP (*r*^2^ > 0.8), suggesting that their observed effect was due to linkage disequilibrium with the top associated SNP. To verify this hypothesis, we reran the association analysis in the INGI-FVG cohort, which showed the strongest association among the samples included in the first-stage meta-analysis (Fig. 3), conditioning on the top SNP (rs78043697). As expected, no significant residual signal remained (*P*-values > 0.05) (data not shown), suggesting that there is only one independent signal in this locus. The phenotypic variance of the 2 kHz hearing explained by rs78043697-C was 1.8%.

**Figure 4.**
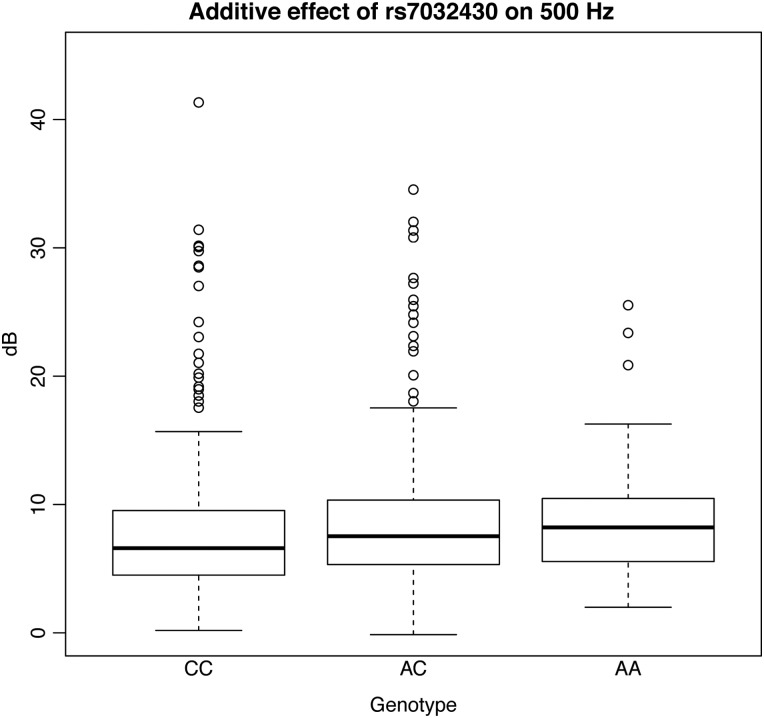
**Boxplot for rs7032430.** The figure displays boxplots for the 500 Hz frequency divided by genotype groups for rs7032430 and adjusted by sex, age and relatedness in our largest cohort (INGI-FVG). As shown in the figure, people carrying the AA genotype have higher values and hence worse hearing function.

**Figure 5.**
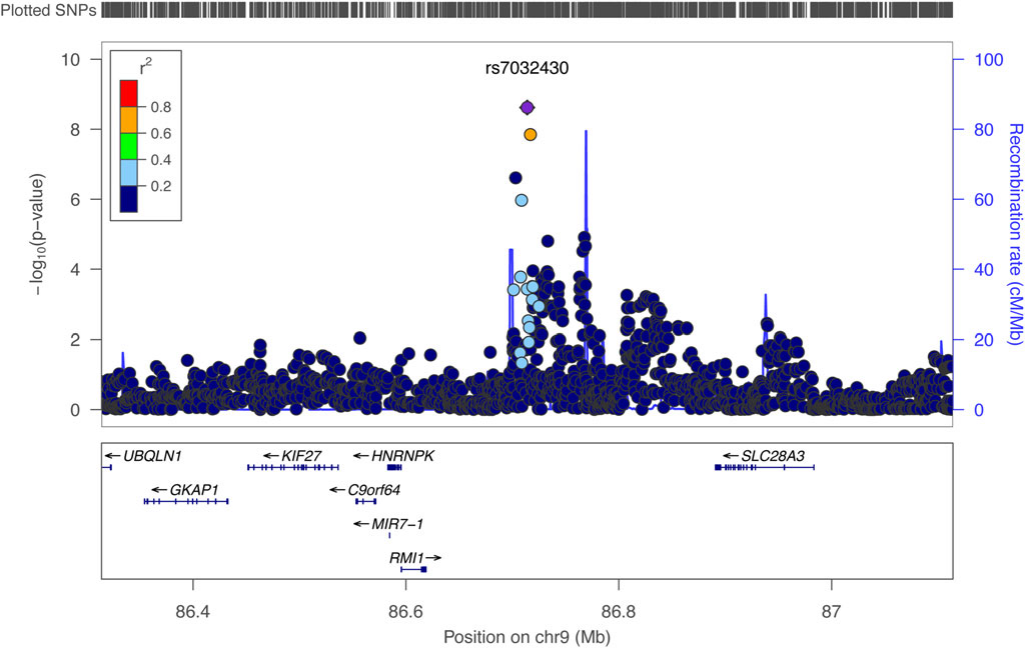
**Locus-zoom plot for chromosome 9 locus.** The plot shows the locus on chromosome 9 associated with normal hearing function measured at the 500 Hz as well as the genes within the region, including *SLC28A3*, the strongest candidate for hearing function. The results plotted were obtained with a two-stage meta-analysis, focusing on Italian isolates first and then including the communities from the Silk Road. The *y*-axis shows −log_10_ of the *P*-values obtained from the complete meta-analysis of the 500-Hz hearing threshold.

The second highest SNP was rs7032430-A (*P* = 2.39E−09 and *P* = 4.22E−09 for 0.5 kHz under additive and dominant models, respectively) located on 9q21.32 and explaining 1.3% of the phenotypic variance of the 0.5 kHz hearing. Subjects carrying the effect allele A (*N* = 1007) have worse hearing compared with those homozygous for the non-effect allele C (*N* = 1622), by ∼1.6 dB in INGI-FVG (Fig. 4). Several SNPs in the same locus show association with the same trait. Among these, rs35664751-A, in high linkage disequilibrium (*r*^2^ > 0.6) with the top SNP (rs7032430-A), reached genome-wide significance (*P*-value = 1.42E−08) (Fig. 5). These SNPs lie close to several genes: *RMI1, HNRNPK, C9orf64, KIF27, GKAP1, UBQLN1* and *SLC28A3*. Prioritization for follow-up studies was based on known functions, belonging to the same gene family as known hearing genes and distance from the associated SNP. Based on these criteria, *SLC28A3* emerged as the strongest candidate, belonging to the ‘solute carriers family’ whose members are already known to be involved in hearing.

Finally, rs2243805-A on 2q14.3, located within *LIMS2* and *GPR17*, reached a genome-wide significant *P*-value of 1.74E−08 at 4 kHz under the dominant model. As no additional evidence of association was found in this region, this SNP was not further studied.

Table 1 shows summary statistics for the above-mentioned loci, whereas Table 2 displays imputation quality (IMPUTE2 info score) across cohorts for the top SNPs. Supplementary Material, Table S3 shows the results (*P* < 0.05) for the top SNPs across all the traits and genetic models. In particular, it can be observed that associations are not limited to a single trait e.g. rs7032430-A is associated (although not significantly) with all the low-medium frequencies (0.25, 0.5, 1 kHz) and to the relative PTAL and PTAM. This is probably due to a true SNP effect on this range of frequencies but also reflects the correlation between the traits. To further prove the validity of our findings, a replication step was performed using the B58C cohort (*N* = 5892), with only two traits available (1 and 4 kHz), and the Finnish Twin Study on Ageing (FITSA) cohort (*N* = 270) from Finland. Among the 35 SNPs, 4 SNPs within the 13q21.31 locus were nominally replicated (*P* < 0.05) at the 1 kHz frequency in the B58C cohort (Table 3). For the 9q21.32 locus, the second best hit rs35664751-A was also nominally replicated (*P* = 0.02761) in the FITSA cohort (Table 3).

**Table 1.** Top SNPs for each locus resulting from the two-step meta-analysis on normal hearing function across six different frequencies (0.25, 0.5, 1, 2, 4 and 8 kHz) and under four genetic models (additive, recessive, dominant and overdominant), including only Italian cohorts in the first step and then the Silk Road cohort in the second step

SNP	Trait and model	Chr	Position	EA	AA	*N*	Beta	SE beta	Final *P*-value	Dir	N. pop.	Orig. p
rs78043697	2 kHz dom	13	62467039	C	T	2155	0.119997443	0.019266322	4.71E−10	+++?+	4	1.83E−08
rs7032430	0.5 kHz add	9	86714002	A	C	2629	0.035277526	0.00591061	2.39E−09	+++++	5	1.27E−08
rs2243805	4 kHz dom	2	128407499	G	A	2632	0.068598345	0.012170387	1.74E−08	+++++	5	6.90E−08
rs35664751	0.5 kHz add	9	86717006	G	A	2630	0.035460097	0.006253427	1.42E−08	+++++	5	2.06E−07

SNP, single nucleotide polymorphism; add, additive; dom, dominant; Chr, chromosome; EA, effect allele; AA, alternative allele (non-effect allele); *N*, number of samples; Beta, beta coefficients; SE, standard error; Dir, direction; +, positive effect; −, negative effect; ?, SNP not present/analyzed (cohorts: INGI-FVG, INGI-CARL, CILENTO, TALANA, SR); N. pop, number of populations; Orig. p, original *P*-value (first-stage meta-analysis).

**Table 2.** Imputation quality score across cohorts for the two best hits

COHORT	INFO rs78043697	INFO rs7032430
INGI-FVG	0.996	0.825
INGI-CARL	0.995	0.766
TALANA	NA	0.795
CILENTO	0.994	0.737
SR	0.998	0.998

INFO, Imputation Info Score (IMPUTE2).

**Table 3.** Association replication results in two independent cohorts from UK and Finland, replicating several SNPs from both candidate loci (*PCDH20* on chromosome 13 and *SLC28A3* on chromosome 9) at a nominal significance level (*P* < 0.05)

SNP	Trait and model	EA	AA	*N*	Beta	SE beta	*P*	Cohort
rs111887296	1 kHz add	T	C	5892	−0.0109	0.005435	0.04495	B58C
rs113274536	1 kHz add	T	C	5892	−0.0111	0.005431	0.04072	B58C
rs75281867	1 kHz add	C	T	5892	−0.0110	0.005432	0.04215	B58C
rs77604362	1 kHz add	T	C	5892	−0.0110	0.005433	0.04239	B58C
rs35664751	0.5 kHz add	G	A	266	0.1017	0.046187	0.02761	FITSA

SNP, single nucleotide polymorphism; add, additive; EA, effect allele; AA, alternative allele; *N*, number of samples; Beta, beta coefficients; SE, standard error; P, *P*-value.

### Sequencing analysis

The top genes located on chromosomes 13 and 9 (*PCDH20* and *SLC28A3*, respectively) were selected for further analyses. To investigate any functional genetic variants marked by the associated tag SNPs (rs78043697 for *PCDH20* and rs7032430 for *SLC28A3*) that contribute to the variation in this trait, a pool of 48 people (24 individuals for each gene) from the INGI-FVG cohort were sequenced. Nine variants within the *PCDH20* gene (3 in 3′ UTR; 5 exonic, of which 2 are synonymous, 2 non-synonymous and 1 frameshift; and 1 in 5′ UTR) and 42 within *SLC28A3* (1 downstream; 18 in 3′ UTR; 5 exonic, of which 2 synonymous and 3 non-synonymous; 14 intronic; and 4 in 5′ UTR) were identified. However, none of the variants identified was associated with the selected genotypes (i.e. tag SNPs). These findings further strengthen the possible presence of variants in the regulatory regions as expected for a quantitative trait. In this light, we consulted RegulomeDB (http://regulomedb.org/), which is a database annotating SNPs for known and predicted regulatory functions, including data from ENCODE, but also other databases and published literature (7). The outcome reported that several SNPs associated with *PCDH20* and *SLC28A3* were located in sites where different transcription factors bind (Supplementary Material, Table S4).

### Gene expression analysis

**Figure 6.**
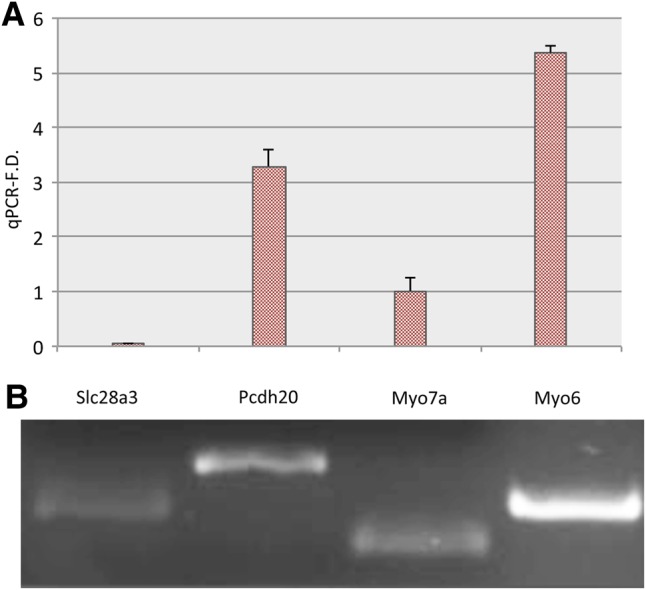
**(A) qReal-time analysis of the selected genes (*PCDH20, SLC28A3*). (B) Semi-quantitative RT-PCR.** The plot shows expression for *Pcdh20* and *Slc28a3*, compared with *Myo7a* and *Myo6* (A). The *y*-axis represents a scale whose unit is the expression of the reference gene *Myo7a. Pcdh20* displays a strong expression, whereas *Slc28a3* shows lower levels. Semi-quantitative RT-PCR demonstrates that both genes are expressed (B).

In order to examine whether *Pcdh20* and *Slc28a3* are expressed in the mouse cochlea, we dissected mouse inner ear tissues at postnatal day 5 (P5) and performed RNA extraction for quantitative RT-PCR (qRT-PCR) and semi-quantitative RT-PCR (sqRT-PCR) (Fig. 6A and B). As positive controls we used both *Myo7a* and *Myo6*, genes required for hair cell differentiation and associated with hereditary deafness (8–10), which are abundantly expressed in inner ear hair cells. Both *Pcdh20* and *Slc28a3* transcripts were amplified by the qRT-PCR and sqRT-PCR, confirming that the two genes are expressed in cochlea (Fig. 5). In particular, *Pcdh20* showed lower expression than *Myo6* and a 3-fold higher expression level than *Myo7a* (Fig. 6A). *Slc28a3* displayed lower expression than these reference genes but was clearly visible on an agarose gel after 30 cycles of sqRT-PCR (Fig. 6B).

**Figure 7.**
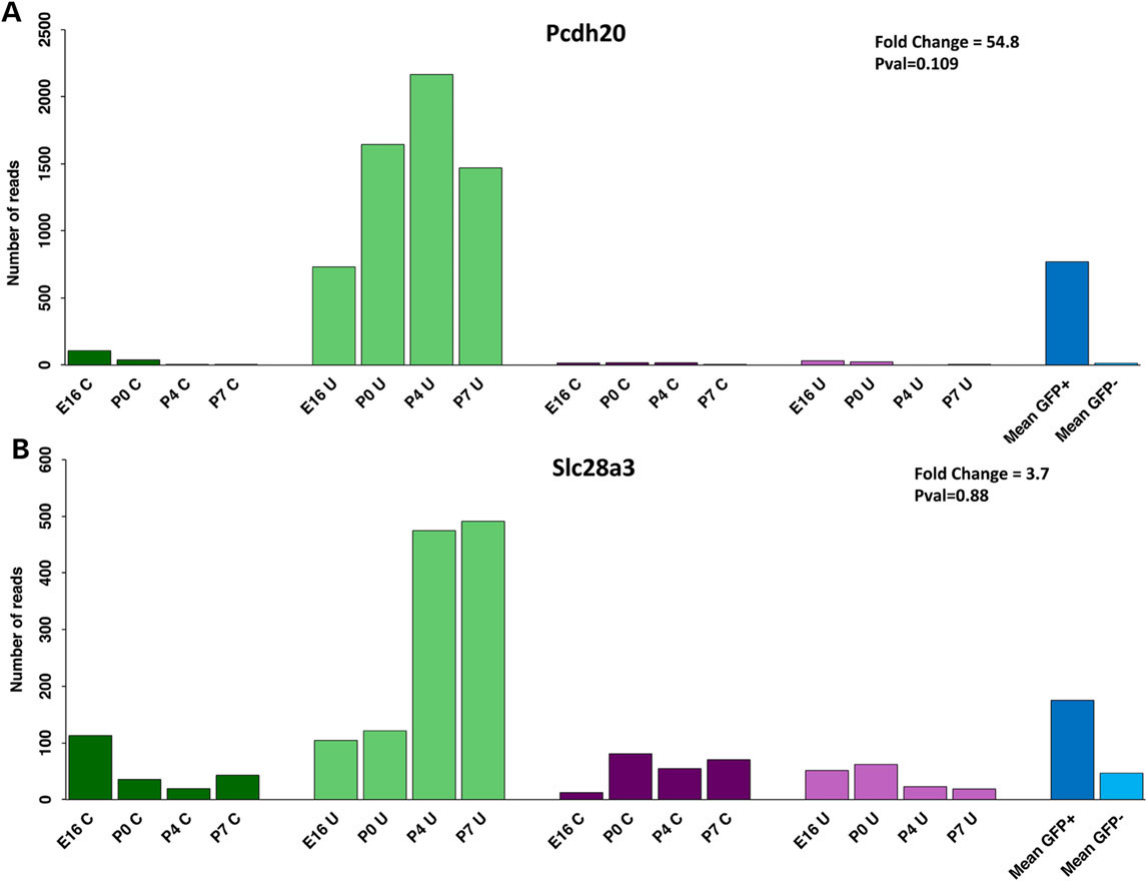
**RNA-seq expression profiles for *PCDH20* (A) and *SLC28A3* (B).** Histograms display the normalized number of reads in hair cells (green) and surrounding cells (purple), in samples from the cochlea (C; dark colors) and utricle (U; light colors) at E16, P0, P4 and P7. The fold change (FC) representing the GFP+/GFP− counts ratio and the multiple test adjusted false discovery rate by the Benjamini–Hochberg procedure are indicated.

RNA-Seq analysis of FACS-sorted cells in the developing mouse cochlea and utricle demonstrated expression of both genes from embryonic day E16 to postnatal day P7 (Fig. 7A and B). Both are enriched in hair cells (GFP+; green) compared with the surrounding cells (GFP−; purple). The overall fold change (blue) shows that *Pcdh20* is highly enriched in hair cells, and *Slc28a3* moderately enriched. Most striking is the strong expression in the utricle, especially at postnatal ages, but there is significant expression in cochlear hair cells as well. In summary, both genes are expressed in the mouse inner ear, with high enrichment in vestibular hair cells and a less restrictive expression pattern in the cochlea.

## Discussion

Genes involved in hearing loss have been identified through many different techniques, beginning with positional cloning and linkage studies. In the past few years, high-throughput and ‘omics’ technologies, including GWAS and Next Generation Sequencing studies, have led to the identification of new genes/mutations in both Mendelian and complex forms of hearing loss. Despite these successes, there is a need for a better understanding of the molecular bases of hearing in complex forms, and as a quantitative trait. In this field, identification of the association of candidate genes at genome-wide statistically significant *P*-values, replication in independent cohorts and demonstration of expression in the inner ear can suggest a fundamental role for the gene products in the auditory system (11).

Following this strategy, we performed a GWAS meta-analysis followed by replication in several cohorts of European and Central Asian ancestry.

This is the largest collaborative effort established on hearing function so far, with a final sample size of 8707 samples and the first GWAS approach to be reported on data imputed to the 1000 Genomes reference panel, resulting in a dense marker map and increased statistical power and precision (12). Although some of the cohorts used here were previously involved in GWAS meta-analysis (3,5), the present study design was strictly focused on Italian cohorts in the discovery phase and involved an overall increased sample size. In this light, it was not surprising that previously reported results were not replicated in the present study. The two-stage meta-analysis approach led to the discovery of two genome-wide significant loci (close to *PCDH20* and *SLC28A3*). This approach was first proposed in order to minimize the required sample size and genotyping without losing power (13) and has been successfully applied in different studies (14–16). It has been argued that this is an efficient way of performing a single study and not a proper replication step (17). Thus, we sought further nominal replication and we successfully replicated both loci in independent cohorts, implying that, despite genetic heterogeneity of the study populations, genes with global importance can be found.

The analysis of individual frequency thresholds as well as the PTAs allowed us to comprehensively portray the normal hearing function. Although multiple phenotypes were tested, these measurements are not independent, thus reducing the concern about multiple testing as already previously discussed (4). Statistical power was also increased by testing four different genetic models, a step of fundamental importance when sample size is limited (18).

The top associated SNPs for the detected loci explained 1.3 and 1.8% of the phenotypic variance. These data show that the associating variants had relatively large effect sizes when compared with other known associations, such as height-associated markers with variance explained ranging from 0.04 to 1.13% (19). We acknowledge the possible limitation of the present study in detecting lower effect variants owing to lack of power. In this light, larger studies could be useful in the future to overcome this problem.

The top genes identified in this study (*PCDH20* and *SLC28A3*) are both members of gene families already implicated in the inner ear. *PCDH20* belongs to the cadherin superfamily, whose members have multiple roles in cellular adhesion (20,21) and have been previously implicated in murine hearing loss as well as in the human Usher syndrome (22). Although very little is known about *PCDH20*, a phylogenetic grouping based on N-terminal sequence suggests that has potential for trans-homotypic and heterotypic interactions (21). We reported RNA-Seq and qRT-PCR for validation of *PCDH20* expression, finding it to be expressed especially in the sensory hair cells. This gene is also reported by the GENSAT atlas as expressed in the spiral ganglion of the inner ear at E15.5 (http://www.gensat.org/index.html).

*SLC28A3* is a member of the solute-carrier family and is thought to concentrate nucleosides including cytidine, uridine, adenosine and guanosine in a cell by coupling translocation of two H^+^ or Na^+^ ions per nucleoside. Its apical location in absorptive epithelia drives vectorial flux of nucleosides, which are essential for nucleotide biosynthesis (23). Three other members of the broad solute-carrier family are implicated in inherited deafness (24–26). Both qRT-PCR and RNA-Seq showed clear expression of SLC28A3 in mouse cochlea and utricle. Moreover, GTEx (http://www.gtexportal.org/) eQTL database revealed a significant association between the top SNP rs78043697-C and *SLC28A3* expression in stomach tissue. These findings support the hypothesis that rs78043697-C could affect the expression of *SLC28A3*, although eQTL data for the inner ear tissue were not available.

Although *SLC28A3* is our strongest candidate, six other genes (*RMI1, HNRNPK, C9orf64, KIF27, GKAP1* and *UBQLN1*) located close to the associated SNP on chromosome 9 were detected. For these genes, a search of published material, existing public databases on gene function, gene expression, related diseases, etc. did not detect any possible link to hearing function.

No causal alleles were identified in the sequencing of the coding regions of *PCDH20* and *SLC28A3*, supporting the involvement of regulatory regions underlying these associations. Such a mechanism is common in GWAS studies, and it has been recently addressed by combining sequencing, epigenetic and transcription-factor data sets (27), which could be a follow-up of the present study.

Finally, it should be noted that *SLC28A3* is directly involved in the metabolism of two drugs (anthracycline and gemcitabine) and in the corresponding side effects/adverse reactions, which also include hearing loss (28,29).

In conclusion, this study reports the discovery of two new genes significantly associated with auditory function in humans and hypothesizes a specific role in the sensory epithelium of the utricle and cochlea. Although this should be confirmed with further experiments, these results increase our knowledge of the molecular basis of normal hearing function by identifying new important candidates for further investigation.

## Materials and Methods

### Sample collection, preparation and genotyping

All studies had ethical approval obtained from the Institutional Review Board of IRCCS—Burlo Garofolo, Trieste, Italy and other involved institutions. Consent forms for clinical studies were signed by each participant, and all research was conducted according to ethics standards defined by the Helsinki declaration. Detailed information about each cohort is shown in Table 4.

**Table 4.** Description of the analyzed cohorts reporting information about sample size, genotyping, filtering and imputation, as well as the different analyses performed

Cohort name	First-step meta-analysis				Second-step meta-analysis	Independent replication	Independent replication
	INGI-FVG	INGI-CARL	TALANA	CILENTO	SR	B58C	FITSA
*N*	980	262	480	433	481	5892	270
DNA collection	Blood	Blood	Blood	Blood	Saliva	Blood and saliva	Blood
SNP chip	Illumina 370 K	Illumina 370 K	Affymetrix 500 K	Illumina 370 K	Illumina 700 K	Illumina Hap550	HumanExome Chip
Genotype calling software	BeadStudio	BeadStudio	Illumina	Illumina	BeadStudio	Mixed	GenomeStudio
Genotype filtering exclusion criteria	MAF < 0.01	MAF < 0.01	MAF < 0.01	MAF < 0.01	MAF < 0.01	MAF < 0.01	MAF < 0.01
	Call rate < 0.97	Call rate < 0.97	Call rate < 0.95	Call rate < 0.95	Call rate < 0.97	Call rate < 0.95	Call rate < 0.95 (<0.99 for SNPs with MAF < 0.05)
	HWE exact test *P* < 1e−08	HWE exact test *P* < 1e−08	HWE exact test *P* < 1e−06	None	HWE exact test *P* < 1e−08	HWE exact test *P* < 1e−04	HWE exact test *P* < 1e−06
Imputation filtering exclusion criteria	MAF < 0.01	MAF < 0.01	MAF < 0.05	MAF < 0.05	MAF < 0.01	MAF < 0.01	MAF < 0.01
	Info score < 0.4	Info score < 0.4	Info score < 0.4	Info score < 0.3	Info score < 0.4	Info score < 0.3	Info score < 0.3
Total SNPs after imputation	8455987	6490547	38020975	6302431	6664949	9031685	9069626
Age range	18–89	18–89	18–92	18–86	18–82	44–45	63–76
Sex (% females)	60	57	56	55.19	58	51	100

#### Italian isolated populations

Altogether 2155 subjects from 4 different cohorts (INGI-FVG, INGI-CARL, Talana and Cilento) from several isolated communities located in Northeast Italy, South Italy and Sardinia were recruited. The collection of genotype and phenotype data followed the protocols previously described (3). In brief, genomic DNA was extracted from blood, using a phenol-chloroform extraction procedure. DNA samples were genotyped with Illumina 370-K and Affymetrix 500-K chips. Quality control filters were applied in each cohort singularly taking into account standard parameters, such as minor allele frequency (MAF), call rate and Hardy–Weinberg equilibrium (Table 4). Finally, all genotypes were checked to ensure that they were reported with the coordinates of the 1000 Genomes Project (NCBI Build 37) reference data and on the forward strand (30). These samples were previously used in GWAS studies (3,5) with another imputation version (HapMap CEU SNP set v22).

#### Silk Road (SR) cohort

We collected samples from 481 people from several rural communities located along the Silk Road, during the Marco Polo Scientific Expedition (31) (see Supplementary Material, Fig. S1). Saliva samples were collected using the Oragene kit (DNA Genotek, Inc.) and DNA extracted according to the supplier's protocols. As for the Italian isolates cohorts, SNP quality filters were applied and coordinates aligned to the forward strand of the reference (releases build 37) (30). These samples were previously used in a GWAS (5) with another imputation version (HapMap CEU SNP set v22).

#### 1958 UK Birth Cohort (B58C)

The B58C and the collection of hearing data have been described previously (6,32). In brief, participants were drawn up from 17 638 individuals born in England, Scotland and Wales within 1 week of March 1958. Of the original cohort, 9377 members were revisited by a research nurse for a biomedical follow-up in 2002–2004. The hearing measure consisted of pure-tone audiometry at 1 and 4 kHz at age 44–45 years and was adjusted for sex, conductive loss, hearing loss in childhood and nuisance variables (noise at test, nurse performing test and audiometer used in test). Genome-wide data for the 1958BC were obtained through several sub-studies, using them as population controls and genotyping a total of 6099 individuals (for details, see http://www2.le.ac.uk/projects/birthcohort/1958bc/available-resources/genetic). These include the Welcome Trust Case Control Consortium study (WTCCC1 and 2) (33), the Type 1 diabetes genetics consortium (T1DGC) (34) and the GABRIEL Large-Scale Genome-Wide Association Study of Asthma (35).

#### The Finnish Twin Study on Ageing

The FITSA cohort includes 217 female twin pairs, aged 63–76 years, recruited in order to investigate the contribution of genetic and environmental factors to the disablement process, and to investigate the effects of ageing on several physiological and functional traits (36). In the current study, a total of 270 subjects from 196 twin pairs with hearing phenotype, covariates and GWAS data available were included in the analysis (91 subjects from 91 monozygotic pairs and 179 subjects from 105 dizygotic pairs, of which 74 included both co-twins and 31 included only one co-twin).

### Imputation and filtering

In order to obtain a homogenous set of markers among all the cohorts and to increase the statistical power, genotypes were imputed following the same protocol in each cohort. In particular, SHAPEIT2 (37) was used for the phasing step and IMPUTE2 for the imputation step to the 1000-Genomes phase I v3 reference set (38). After imputation, SNPs with MAF of <0.01 or imputation quality score (Info) of <0.4 were excluded from the statistical analyses. Table 4 shows the final number of SNPs analyzed after imputation and filtering.

### Audiometric evaluation

Audiometric tests and a careful clinical examination (psychological, neurological, cardiological evaluations, etc.) were carried out for each individual of each cohort. Thresholds for six different frequencies (0.25, 0.5, 1, 2, 4 and 8 kHz) were measured, with exception of the B58C cohort, where only 1 and 4 kHz were available. Three pure-tone averages (PTAs) of air-conduction thresholds were calculated: PTAL at low frequencies (0.25, 0.5 and 1 kHz), PTAM at middle frequencies (0.5, 1 and 2 kHz) and PTAH at high frequencies (4 and 8 kHz). Overall, nine hearing traits were analyzed. Moreover, to avoid non-genetic variations in the hearing phenotype (e.g. unilateral hearing loss), the best hearing ear was chosen. Familial forms of inherited hearing loss were excluded from the study, as well as subjects affected by diabetes or other systemic disorders producing hearing loss.

### Association analysis

Genome-wide association studies were separately performed in each cohort, testing individual hearing thresholds and the PTAs (after adjustment for sex and age) in a linear mixed model in order to account for genomic kinship. Four different genetic models were tested: additive, dominant, recessive and overdominant. All the analyses were carried out using the GRAMMAR-Gamma method as implemented in the GenABEL suit (39) for genotyped SNPs and MixABEL (40) for imputed data. We did not consider any *P*-value correction for the different genetic models tested because these are not four independent tests (41).

In order to increase the statistical power, a two-stage meta-analysis approach was used. The first stage included only Italian cohorts, using a fixed-effects meta-analysis with inverse variance weights as implemented in METAL software (42). Resulting SNPs with *P*-value of <1e−06 were carried out by joining the SR cohort and repeating the analysis (second stage). The final significance level was set to 5e−08.

Finally, following the same analysis protocol, two independent cohorts (B58C and FITSA) were investigated for additional replication, at a nominal level of significance (*P*-value < 0.05).

For each GWAS and meta-analysis, population stratification was checked by computing the lambda coefficient of inflation. The phenotypic variance explained by single SNPs was computed from the mixed-model regression score estimates.

### RNA extraction, reverse transcriptase–polymerase chain reaction (RT-PCR) and real-time quantitative RT-PCR

Total RNA was isolated from postnatal day 5 (P5) mouse cochlea, extracted with a High Pure RNA Isolation Kit (Roche) and quantified on a Nanodrop ND-1000 spectrophotometer (NanoDrop Technologies, Wilmington, DE, USA). Total RNA was used for cDNA synthesis using the Transcription First Strand cDNA Synthesis Kit (Roche, Germany). Aliquots (1 μl) of the RT products were subsequently used for PCR amplification. PCR reactions were optimized to 95°C for 2 min, 30 amplification cycles at 95°C for 10 s, 50°C for 15 s, 72°C for 4 s and a final extension of 1 min at 72°C using Kapa HiFi HotStart ReadyMix PCR kit (Kapa Biosystem, Cape town, South Africa). Amplified products were resolved on 3% agarose gels and visualized by ethidium bromide staining.

Quantitative Real Time PCR (qRT-PCR) for Slc28a, Pcdh20, Myo7a and Myo6 genes was performed using standard PCR conditions in a 7900HT Fast Real Time PCR (Life Technologies) with Power SYBR Green PCR Master Mix (Life Technologies). Gene-specific primers were designed by using PRIMEREXPRESS software (Applied Biosystems). The thermal cycling conditions were an initial denaturation step at 95°C for 10 min, 40 cycles at 95°C for 15 s, 59°C for 1 min and 72°C for 30 s. The experiments were performed in biological triplicates.

#### RNA-Seq expression profiling

We retrieved RNA-Seq data from the Shared Harvard Inner-Ear Laboratory Database (SHIELD), https://shield.hms.harvard.edu/ (43).

### Sequencing

We selected a pool of 48 subjects from the INGI-FVG cohort for sequencing the candidate genes. The subjects were divided in four groups based on their genotypes at the top two associated SNPs. In particular, following the genetic models of the associations detected, the groups consisted of alternative homozygotes versus reference homozygotes and/or heterozygotes for each allele (see Results). For the *PCDH20* gene, the selection was done according to the dominant model of association as follows: (a) 12 carriers of the effect allele of rs78043697 (CC or CT) and (b) 12 homozygotes for the non-effect allele (TT). For the *SLC28A3* gene, the selection was done under the additive model as follows: (c) 12 homozygotes for the effect allele of rs7032430 (AA) and (d) 12 homozygotes for the non-effect allele (CC). The coding sequence and the intron-exon boundaries were analyzed by Sanger sequencing. A standard PCR was carried out at 60°C, for 35 cycles using KAPA2G Fast ReadyMix PCR Kit (Kapa Biosystems), according to the manufacturer's protocol. Sequencing was performed on a 3500Dx Genetic Analyzer (Life Technologies, CA, USA), using 3.1 Big Dye terminator chemistry (Life Technologies, CA, USA) according to the manufacturer's instructions. Primer sequences are available upon request.

## Supplementary Material

Supplementary Material is available at *HMG* online.

## Funding

Research activities have been carried out with funds from the Italian Ministry of Health (RF2010 to P.G.), RC2008 to P.G. and NIH DC02281 to D.P.C. Phenotyping and genotyping of the FITSA sample was supported by the Academy of Finland Center of Excellence in Complex Disease Genetics (grants 213506 and 129680), the Academy of Finland (grants 100499, 205585, 118555, 141054, 265240, 263278 and 264146 to J.K., grant 69818 to T.R., grants 251723 and 263729 to A.V.), Sigrid Juselius Foundation (to J.K.) and the Welcome Trust Sanger Institute, UK. For the B58C cohort, this work was funded under the following: the Haigh Fellowship in age-related deafness, Deafness Research UK (444:UEI:SD), Medical Research Council grant G0000934 and the Wellcome Trust grant 068545/Z/02. Genotyping for the B58C-WTCCC subset was funded by the Wellcome Trust grant 076113/B/04/Z. The B58C-T1DGC genotyping utilized resources provided by the Type 1 Diabetes Genetics Consortium, a collaborative clinical study sponsored by the National Institute of Diabetes and Digestive and Kidney Diseases (NIDDK), National Institute of Allergy and Infectious Diseases (NIAID), National Human Genome Research Institute (NHGRI), National Institute of Child Health and Human Development (NICHD) and Juvenile Diabetes Research Foundation International (JDRF) and supported by U01 DK062418. B58C-T1DGC GWAS data were deposited by the Diabetes and Inflammation Laboratory, Cambridge Institute for Medical Research (CIMR), University of Cambridge, which is funded by Juvenile Diabetes Research Foundation International, the Wellcome Trust and the National Institute for Health Research Cambridge Biomedical Research Centre; the CIMR is in receipt of a Wellcome Trust Strategic Award (079895). The B58C-GABRIEL genotyping was supported by a contract from the European Commission Framework Programme 6 (018996) and grants from the French Ministry of Research.

## Sites

http://hereditaryhearingloss.org/; http://www2.le.ac.uk/projects/birthcohort/1958bc/available-resources/genetic; http://www.gtexportal.org/; http://www.gensat.org/index.html; http://omim.org/; http://www.ncbi.nlm.nih.gov/pubmed; https://dnanexus.com/; https://shield.hms.harvard.edu/; http://regulomedb.org/.

## Supplementary Material

Supplementary Data
