# Supplementary material for: Genome-wide association analysis on normal hearing function identifies PCDH20 and SLC28A3 as candidates for hearing function and loss
Source: Hum Mol Genet. 2015 Jul 17;24(19):5655–64. doi: 10.1093/hmg/ddv279 (PMC4572074; doi:10.1093/hmg/ddv279)
Supplement: Supplementary Data [file supp_24_19_5655_v2_index.html]

Genome-wide association analysis on normal hearing function identifies PCDH20 and SLC28A3 as candidates for hearing function and loss — Genome-wide association analysis on normal hearing function identifies PCDH20 and SLC28A3 as candidates for hearing function and loss — Supplementary Data 

# Genome-wide association analysis on normal hearing function identifies *PCDH20* and *SLC28A3* as candidates for hearing function and loss

## Supplementary Data

Supplementary Data

- Supplementary File 1 - zip file
- Supplementary File 2 - zip file
- Supplementary Table 1 - xlsx file
- Supplementary Table 2 - xlsx file
- Supplementary Table 3 - xlsx file
- Supplementary Table 4 - xlsx file
- Supplementary File 3 - zip file
